# A Dietary Sugarcane-Derived Polyphenol Mix Reduces the Negative Effects of Cyclic Heat Exposure on Growth Performance, Blood Gas Status, and Meat Quality in Broiler Chickens

**DOI:** 10.3390/ani10071158

**Published:** 2020-07-08

**Authors:** Majid Shakeri, Jeremy J. Cottrell, Stuart Wilkinson, Hieu H. Le, Hafiz A. R. Suleria, Robyn D. Warner, Frank R. Dunshea

**Affiliations:** 1 Department of Medicine, The University of Washington, Seattle WA 98102, USA; 2 Faculty of Veterinary and Agricultural Sciences, The University of Melbourne, Parkville Melbourne, Victoria 3010, Australia; huul1@student.unimelb.edu.au (H.H.L.); hafiz.suleria@unimelb.edu.au (H.A.R.S.); robyn.warner@unimelb.edu.au (R.D.W.); fdunshea@unimelb.edu.au (F.R.D.); 3 Feedworks Pty Ltd., Romsey, Victoria 3434, Australia; stuart.wilkinson@feedworks.com.au; 4 Faculty of Biological Sciences, The University of Leeds, Leeds LS2 9JT, UK

**Keywords:** heat stress, polyphenols, growth rate, meat quality, hormones, broiler chickens

## Abstract

**Simple Summary:**

Heat stress is a main reason of systemic oxidative stress, which compromises broiler meat production and quality. To improve the productivity of poultry meat production, studies have investigated different heat stress amelioration strategies. Among these strategies, low-cost feed supplementations are introduced to potentially reduce the negative effects of heat stress. Previous studies have also investigated the effects of different antioxidants on growth performance and meat quality, while a limited number of studies have been made regarding the impacts of the polyphenols at different doses. Polyphenols with antioxidant properties have positive effects against oxidative stress, and are naturally available in high amounts in plants, which makes them a novel feed supplementation for improving meat production as well as meat quality in heat-stressed broiler chickens. Therefore, this study attempted to investigate the effects of different doses of polyphenols supplementation on growth performance, physiological responses, and meat quality in broiler chickens exposed to cyclic heat stress.

**Abstract:**

Heat stress (HS) compromises growth performance and meat quality of broiler chickens by interrupting lipid and protein metabolism, resulting in increased oxidative damages. The experiment attempted to investigate whether dietary polyphenols (Polygain (POL)) could ameliorate the aforementioned adverse effects of HS on performance and meat quality. One hundred and twenty one day-old-male chicks were allocated to two temperature conditions, thermoneutral (TN) or HS, and fed with either a control diet (CON) or the CON plus four different doses of POL (2, 4, 6 and 10 g/kg). Heat stress caused respiratory alkalosis as evidenced by increased rectal temperature (*p* < 0.001) and respiration rate (*p* < 0.001) due to increased blood pH (*p* < 0.001). Heat stress decreased final body weight (*p* = 0.061) and breast muscle water content (*p* = 0.013) while POL improved both (*p* = 0.002 and *p* = 0.003, respectively). Heat stress amplified muscle damages, indicated by increased thiobarbituric acid reactive substances (*p* < 0.001) and reduced myofibril fragmentation index (*p* = 0.006) whereas POL improved both (*p* = 0.037 and *p* = 0.092, respectively). Heat stress impaired meat tenderness (*p* < 0.001) while POL improved it (*p* = 0.003). In conclusion, HS impaired growth performance and meat quality whereas POL ameliorated these responses in a dose-dependent manner, and effects of POL were evident under both temperature conditions.

## 1. Introduction

Heat stress (HS) has undesired effects on the growth performance [1,2] and meat quality [3] of broiler chickens. During *HS*, chickens seek to dissipate unwanted and excess body heat by increasing panting, resulting in alteration of blood acid/base status, which in turn can give rise to *respiratory alkalosis* [4] and disruptions in muscle membrane integrity [3]. *Additionally*, the work required to maintain body temperature consumes diverts energy from metabolic pathways resulting in reduced performance and impaired eating quality [5]. Furthermore, HS results in oxidative stress and membrane lipid oxidation leading to unfavourable meat colour and flavour [6] and impacting on muscle fibre structure [7,8]. To mitigate the unfavourable impact of HS on meat quality and performance, a number of nutritional approaches to decrease oxidation have been suggested [9].

Polyphenols are a group of compounds with antioxidant properties that can be found in various part of vegetables, fruits including roots, leaves and flowers [10,11] agri-food waste and by products [11]. Polyphenols have anti-inflammatory and immunomodulatory properties that prevent oxidative stress [10,12] by reducing production of reactive oxygen species. Also, polyphenols can act as hormones and growth regulators [13] and enzymes modulators [14]. Therefore, supplemental dietary polyphenols may ameliorate the negative effects of HS on performance and meat quality.

Recently, a sugar cane molasses extract that is rich in polyphenols as well as a number of apigenin-, methoxyluteolin- and tricin-O- glycosides has been characterised [15]. It was hypothesised that dietary supplementation of this molasses extract may reduce oxidative stress in HS broilers and ameliorate some of the negative effects on growth and meat quality.

## 2. Materials and Methods

### 2.1. Ethics Statement

The experiment was approved by The University of Melbourne (FAVS, Protocol no. 18144261.1).

### 2.2. Animals, Experimental Design and Diets

On hundred and twenty one day-old male Ross-308 chicks obtained from a local hatchery (Tri Foods Pty. Ltd., Bannockburn Victoria, Australia) were transported for 2 h while being maintained at ~30 °C to the research the site located in The University of Melbourne. Upon arrival the chicks were wing-tagged, individually weighed and randomly allocated to 20 pens in two climate-controlled rooms (three chicks/pen and four replicates for each treatment). There were an additional four pens/temperature room of chicks from the same hatching in each room that were utilised in a concurrent experiment with both experiments utilising the same control diet [16]. The temperature for both rooms was maintained at 33 °C (continuously for the first 7 days) and decreased to 25 °C for TN (thermoneutral) (days 7–35); or HS where the temperature was at 33 °C (8 h/day) and 25 °C (16 h/day) from days 7–35. The relative humidity was maintained at 50 ± 5% throughout the experiment. Light was provided continuously for the first 3 days and was reduced (1 h/day) to 20 h on day 7 for both rooms. Each pen (100 cm × 50 cm) was equipped with a nipple drinker and a feeder and the floor was covered with wood shaving (~10 cm deep).

Chickens were fed either a commercial control diet (CON) or the CON plus 2, 4, 6 or 10 g/kg of the molasses extract (Polygain, The Product Makers, Keysborough, Australia) (POL, Table 1) [15].

Polygain is polyphenol-rich, abundant in minerals, nutrients and amino acids. Commercial feed was provided as a starter (first and second week, 24.9% crude protein and 12.0 metabolic energy/kg), grower (third and fourth week, crude protein 23.1% and 12.4 metabolic energy/kg) and finisher (fifth week, 21.3% crude protein and 12.7 metabolic energy/kg). Chickens had unlimited access to feed and water. Body weight and consumed feed were recorded weekly.

Rectal temperature was measured weekly at three time points (09:00 before increasing the room temperature, 12:00 and 16:00 before the room temperature was decreased) with a thermometer (Comark PDT 300, Norwich, UK). Video recordings (2 chickens/pen) were made at 12:00 on day 34 with a smartphone (iPhone 7, Apple Inc., Cupertino, CA, USA) for subsequent analysis of respiration rate.

Forty chickens (1/pen) were randomly selected for blood analysis (4/treatment) at the end of study. About 3 mL blood was collected from the wing vein and ~0.2 mL was used for blood gas, haematology and electrolyte analysis (EPOC^®^; Alere, Waltham, MA, USA). The remaining blood was centrifuged at 15,000× *g* for 15 min. to obtain plasma for further analysis for metabolite and hormone concentrations.

### 2.3. Slaughter and Objective Meat Quality Measures

Slaughter procedure was performed as detailed in our previously published paper [17]. Briefly, after feather removal, the breast muscles (*m. Pectoralis major and minor*) were partially skinned to measure pH at less than 10 min, 1 h and 24 h after killing by using a digital pH meter (WP-80M, TPS, Pty Ltd., Springwood, Queensland, Australia) that was calibrated at pH 4.00 and 7.00. Carcasses were then rapidly chilled by placing in sealed plastic bags suspended in cold water (~4 °C for 40 min.) before transferring to a refrigerated room (4 °C) to simulate commercial conditions. Other meat quality analysis such as drip loss, cooking loss, shear force and colour were performed as explained in detail in our previously published paper [17].

### 2.4. Biochemical and Hormone Analyses

Muscle myofibrillar fragmentation index (MFI) and thiobarbituric acid reactive substances (TBARS) were determined as described in detail in our previously published paper [17]. Plasma and muscle betaine concentrations were determined using the high-performance liquid chromatography (Waters 2998, Massachusetts, USA) technique as described previously [17]. Plasma thyroid hormone concentrations were measured using free triiodothyronine (T_3_) and thyroxine (T_4_) radioimmunoassay kits (MP Biomedicals, LLC Diagnostics Division, Ohio, USA) based on a protocol provided by the manufacturer [17]. Muscle protein carbonyl concentrations were determined using a method previously published with some modifications [18]. Briefly, muscle (1 g ± 0.01) was minced and homogenised (IKA Ultra Turrax^®^ T 25 digital, Selangor, Malaysia) in 5 mL pyrophosphate buffer (2 mM sodium pyrophosphate, 10 mM tris maleate, 100 mM potassium chloride and 2 mM ethylene tetra acetic acid at pH = 7.4) at 5000× *g* for 30 s. After splitting into 2 tubes, 9 mL hydrogen chloride-acetone (3:100 *v/v*) was added in each tube, vortexed and centrifuged at 5000× *g* for 5 min at 4 °C. The mixture was washed twice with 1 mL trichloroacetic acid 10% and centrifuged at 5000× *g* at 4 °C for 5 min. Tube 1 was then incubated with 0.5 mL 10 mM 2,4 dinitrophenylhydrazine (DNPH) dissolved in 2 mM hydrogen chloride whereas tube 2 was incubated with only 0.5 mL 2 mM hydrogen chloride to calculate protein concentration. Both tubes were kept in darkness for 30 min (vortexed for 10 sec every 10 min). Next, 0.5 mL 20% trichloroacetic acid was added to both tubes which were vortexed and placed on ice for 10 min. before being centrifuged at 5000× *g* for 5 min at 4 °C. After washing with 1 mL trichloroacetic acid 20% the centrifuged pellets were washed three times with 1 mL ethanol ethyl acetate and then centrifuged at 5000× *g* for 5 min at 4 °C. Next, 1 mL of a guanidine solution (6 M guanidine hydrochloride dissolved in 20 mM potassium dihydrogen phosphate at pH 2.3) was added and the mixture shaken overnight at 4 °C. Absorbance was measured at 370 nm to determine DNPH concentration in tube 1 and at 280 nm to determine protein concentration in tube 2.

### 2.5. Statistical Analyses

All results were analysed with one way ANOVA for the main and interactive effects of temperature (T) and the linear (L) and quadratic (Q) effects of POL using GenStat V.16 (VSNi Ltd., Hemel Hempstead, UK). For physiological parameters and muscle TBARS the main and interactive effects of time (TM) were also included as repeated measures effects in the residual maximum likelihood model (REML) with pen as random effect. Differences were considered a trend when 0.10 > *p* > 0.05 and significant when *p* < 0.05.

## 3. Results

### 3.1. Body Weight and Feed Consumption

There was no effect of temperature on either average daily feed intake (ADFI), average daily gain (ADG) or feed conversion ratio (FCR) between days 0–21 of the experiment (Table 2). However, between days 21–35 there was a reduction in ADG (90.3 vs. 85.2 g/d, *p* = 0.029) and ADFI (143 vs. 135 g/d, *p* = 0.024) with no change in FCR in response to HS (Table 2). Dietary POL increased ADG (82.1 vs. 85.6, 87.6, 91.5 and 92.0 g/d, *p* = 0.004) and decreased FCR (1.68 vs. 1.65, 1.60, 1.47 and 1.55 g/d, *p* = 0.015) in a linear manner with increasing dose of POL but did not affect ADFI between days 21–35. Over the entire experiment, there tended to be a reduction in ADG (55.9 vs. 53.8 g/d, *p* = 0.060) and ADFI (87.8 vs. 83.3 g/d, *p* = 0.062) with no change in FCR in response to HS (Table 2). Dietary POL increased ADG (52.5 vs. 53.6, 55.3, 54.8 and 57.9 g/d, *p* = 0.002) and decreased FCR (1.60 vs. 1.64, 1.61, 1.52 and 1.50 g/d, *p* = 0.050) in a linear manner with increasing dose of POL but did not affect ADFI over the entire experiment. As a result of the effects on ADG, final live weight tended to be decreased by HS (1994 vs. 1920 g, *p* = 0.061) and increased linearly with increasing dose of POL (1877 vs. 1915, 1972, 1957 and 2065 g, *p* = 0.002).

### 3.2. Rectal Temperature and Respiration Rate

**Figure 1:**
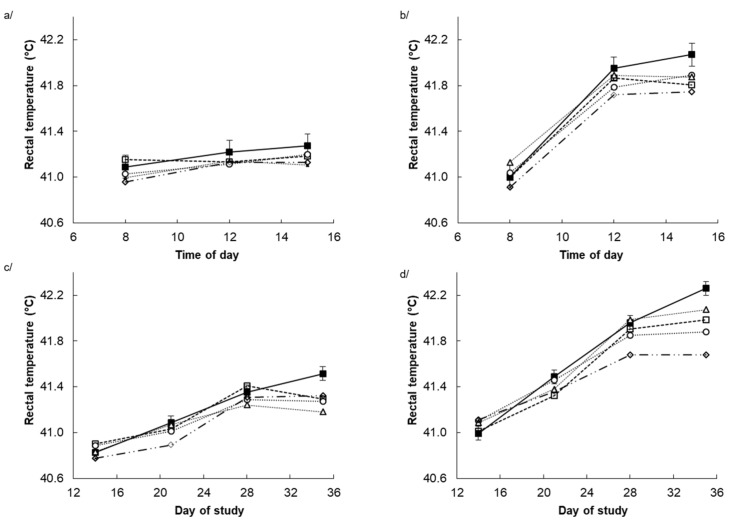
Rectal temperature of chickens supplemented with different doses of polygain (0 g/kg ■, 2 g/kg ∆, 4 g/kg □, 6 g/kg ○ and 10 g/kg ◊ of POL), when chickens exposed to thermoneutral (**a/** and **c/**) or cyclic heat stress (**b/** and **d/**) conditions. Panels a/ and b/ show the effect of time of day (TM) pooled across days of the experiment (DAY) with the standard error of the difference (SED) for the interaction between TM, environmental temperature (T) and diet (D) displayed on the data from the chickens receiving the CON diet. Panels c/ and d/ show the effect of DAY pooled across TM with the SED for the interaction between DAY, T and D displayed on the data from the chickens receiving the CON diet. There were main effects (*p* < 0.10) of T (*p* < 0.001), DAY (*p* < 0.001), TM (*p* < 0.001) and linear effects of POL (L) (*p* < 0.001) and interactive effects of T × DAY (*p* < 0.001), T × TM (*p* < 0.001), DAY × TM (*p* < 0.001), TM × L (*p* < 0.001), DAY × D (*p* < 0.001), DAY × T × L (*p* = 0.008) and T × DAY × TM (*p* < 0.001). There were no other main or interactive effects (*p* < 0.10).

Heat stress significantly increased rectal temperature (*p* < 0.001) with the most significant response (*p* < 0.001) being during the afternoon (Figure 1b) and towards the end of the experiment (Figure 1d). Dietary POL decreased (*p* < 0.001) rectal temperature in a linear fashion (*p* < 0.001) with the response being greatest in those chickens exposed to HS, during the afternoon (Figure 1b) and towards the end of the experiment (Figure 1d) as indicated by T × DAY (*p* < 0.001), T × TM (*p* < 0.001), DAY × TM (*p* < 0.001), TM × L (*p* < 0.001), DAY × L (*p* < 0.001), DAY × T × L (*p* = 0.008) and T × DAY × TM (*p* < 0.001) interactions. Consequently, on the afternoon of day 35 of age HS increased rectal temperature by 1.1 °C in chickens consuming the CON diet (41.55 vs. 42.71 °C) whereas chickens consuming the highest POL diet had a rectal temperature of only 42.01 °C during HS (Table 3). Respiration rate at 11:00 am on day 35 was increased by HS (64 vs. 160 bpm, *p* < 0.001) and was decreased linearly by dietary POL (122 vs. 114, 111, 109 and 106 bpm, *p* = 0.012) (Table 3). However, there was a T × L interaction (*p* = 0.022) such that the increase in respiration rate in response to HS was greater in chickens consuming the CON diet (66 vs. 177 bpm) than in those consuming the highest POL diet (64 vs. 148 bpm) (Table 3).

### 3.3. Blood and Plasma Parameters and Muscle Betaine

Heat stress increased blood pH (7.35 vs. 7.44, *p* < 0.001) and tended to increase linearly with increasing dose of POL (7.36 vs. 7.39, 7.39, 7.40 and 7.42, *p* = 0.068) (Table 3). Blood pCO_2_ was decreased by HS (51.6 vs. 41.1 mm Hg, *p* < 0.001) whereas there was no effect of POL. There was no main effect of T on blood total CO_2_, pO_2_, O_2_ saturation, HCO_3_ and base excess (Table 3). However, there was an interaction (*p* = 0.014) between temperature and a linear effect of POL such that blood total CO_2_ increased with increasing dose of POL under TN conditions but not during HS. There were quadratic effects of POL on blood pO_2_ and O_2_ saturation (*p* < 0.001) in that both were highest at intermediate doses of POL. There was a main linear POL effect (*p* = 0.015) and an interaction (*p* = 0.003) between temperature and a linear effect of POL such that blood HCO_3_ increased with increasing dose of POL under TN conditions but not during HS. Similarly, there was a main linear POL effect (*p* = 0.006) and an interaction (*p* = 0.002) between temperature and a linear effect of POL such that blood base excess increased with increasing dose of POL under TN conditions but not during HS. The blood anion gap was decreased by HS (17.5 vs. 15.5 mM, *p* < 0.001) and decreased linearly with increasing dose of POL (17.9 vs. 17.2, 15.3, 17.2 and 15.2 mM, *p* < 0.001). However, there was an interaction with temperature such that this decrease with increasing dose of POL only occurred under TN and not during HS.

Blood haematocrit tended to be decreased by HS (19.7 vs. 18.9%, *p* = 0.087) and increased quadratically (*p* = 0.042) with dose of POL peaking at 4 g/kg POL (Table 4). There was no effect of T on blood haemoglobin concentrations whereas it tended to increase quadratically (*p* = 0.082) with increasing dose of POL peaking at 4 g/kg POL. Blood potassium (6.40 vs. 5.50 mM, *p* < 0.001) and sodium (150 vs. 148 mM, *p* = 0.001) concentrations were decreased during HS but there was no effect on blood chloride or calcium concentrations (Table 4). While there were no main effects of POL on blood potassium concentrations there was an interaction (*p* = 0.028) such that blood potassium concentrations decreased linearly with increasing dose of POL under TN but not in those exposed to HS conditions. There were linear (*p* = 0.042) and interactive (*p* = 0.056) effects of POL such that blood chloride decreased linearly with increasing dose of POL particularly under TN conditions (Table 4). There were quadratic (*p* = 0.062) and interactive (*p* = 0.035) effects of POL such that blood calcium decreased quadratically before increasing again with increasing dose of POL with the nadir occurring at different doses of POL under TN (2 g/kg) and HS (6 g/kg) conditions (Table 4). Blood lactate concentrations decreased by HS (7.51 vs. 4.77 mM, *p* < 0.001) and decreased linearly (*p* = 0.011) with increasing dose of POL. However, there was an interaction such that blood lactate concentrations decreased with increasing dose of POL under TN conditions whereas they increased with increasing dose under HS conditions. Blood glucose concentrations tended to increase by HS (14.4 vs. 15.0 mM, *p* = 0.058) whereas there was no effect of POL. Although there were no main effects of temperature or POL on plasma T_3_ concentrations there tended to be a T × L interaction (*p* = 0.083) such that plasma T_3_ concentrations were increased with increasing dose of POL under TN conditions not under HS conditions (Table 4). Plasma T_4_ (6.64 vs. 9.27 pg/mL, *p* < 0.001) concentrations were increased by HS but were unchanged by dietary POL. Plasma betaine concentrations were decreased by HS (139 vs. 108 µmol/L, *p* < 0.001) and responded quadratically to increasing dose of POL (118 vs. 132, 129, 121 and 116 µmol/L, *p* = 0.023). Muscle betaine concentrations were decreased by HS (155 vs. 126 µmol/g, *p* = 0.002). There were main and interactive linear (*p* < 0.001, both) and quadratic (*p* = 0.006, both) effects of POL such that muscle betaine concentrations were only lower during HS in the chickens fed the CON (Table 4).

### 3.4. Objective Meat Quality

There was no effect of temperature or dietary POL on breast muscle cooking loss (Table 5). No main effect of HS on drip loss; there were main (*p* = 0.007) and interactive (*p* = 0.018) linear effects of POL such that drip loss decreased with increasing dietary POL content especially under TN conditions. Water content was decreased by HS (77.2 vs. 76.0%, *p* = 0.013) and increased linearly with dietary POL content (75.4 vs. 75.9, 77.8, 76.3 and 77.7%, *p* = 0.003). Shear force increased after HS (20.9 vs. 24.1 N, *p* < 0.001) and decreased linearly in response to dietary POL (24.4 vs. 23.2, 22.1, 21.5 and 21.3 N, *p* = 0.003). The myofibrillar fragmentation index was decreased by HS (93.8 vs. 69.7, *p* = 0.006) and tended to increase linearly with increasing dose of dietary POL (73.5 vs. 64.9, 96.1, 83.5 and 91.0, *p* = 0.092) (Table 5). There was no effect of temperature on carbonyl units which tended to exhibit a quadratic decline with increasing dose of dietary POL (3.50 vs. 3.22, 3.01, 2.87 and 3.12 nmol/mg protein, *p* = 0.078).

There were significant changes in breast muscle colour between 24 h and 72 h post-slaughter with decreases in L * (56.0 vs. 50.8, *p* < 0.001), a * (1.69 vs. 1.34, *p* = 0.009) and b * (6.10 vs. 4.75, *p* < 0.001) (Table 6). Breast muscle L * (52.7 vs. 54.1, *p* = 0.025) was increased and a * (1.85 vs. 1.18, *p* < 0.001) was decreased by HS but were unchanged by dietary POL. While there was no main effect of temperature on b * there were main and interactive effects of dietary POL such that b * increased with increasing dose of POL before declining with the peak value occurring at a lower dose of POL under TN (4 g/kg) conditions during HS (6 g/kg) (Table 6). Breast muscle pH decreased over time post-slaughter (6.86 vs. 6.61 and 5.87 at 10 min., 1 h and 24 h post-slaughter, *p* < 0.001), was decreased by HS (6.50 vs. 6.39, *p* < 0.001) and increased in a linear manner with dose of POL (6.33 vs. 6.40, 6.48, 6.51 and 6.54, *p* < 0.001) (Table 6). However, there were interactions such that the linear effects of POL were most pronounced at 24 h post-slaughter, particularly under TN conditions. In this context, the pH of the breast muscle from chickens consuming a diet containing 10 g/kg POL and maintained under TN conditions was markedly higher than in muscle from those consuming the control diet (5.72 vs. 6.33).

**Figure 2:**
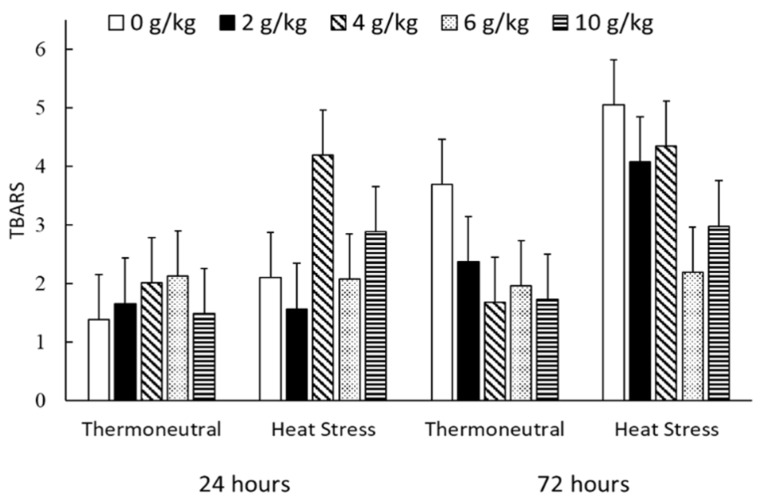
Effect of temperature condition, dose of dietary polygain (POL), and time post-slaughter on breast muscle lipid peroxidase (TBARS) in mg/kg of *malondialdehyde* triangles. Data are mean and standard error of the difference for the interaction between environmental temperature (T), diet (D) and time post-slaughter (TM). There were main effects (*p* < 0.10) of T (*p* = 0.001), TM (*p* < 0.001) and linear (L) effects of POL (*p* = 0.037) and interactive TM × L (*p* < 0.001) and TM × quadratic (*p* = 0.008) effects. There were no other main or interactive effects (*p* < 0.10).

Breast muscle TBARS increased between 24 h and 72 h post-slaughter (2.15 vs. 3.01 mg/kg of *malondialdehyde (MDA)*, *p* < 0.001), was increased by HS (2.01 vs. 3.15 mg MDA/kg, *p* < 0.001) and decreased linearly with increasing dose of dietary POL (3.06 vs. 2.42, 3.06, 2.09 and 2.27 mg MDA/kg, *p* = 0.037) (Figure 2). However, there were significant interactions between time and dose of POL that were largely driven by possibly an anomalous high value at 24 h for samples from chickens fed 4 g/kg and housed under HS conditions (Figure 2). This is particularly so given that there was no further increase between 24 h and 72 h for this group.

## 4. Discussion

The major finding from this experiment was that dietary POL could partially reduce the negative effects of HS on growth performance and meat quality in broiler chickens. The adverse effects of HS on weight gain and meat quality confirm our previous findings [1,17] and others [19,20]. In this regard, potential additives such as POL could play an important role to reduce the negative effects of HS. The effects were such that POL improved body weight gain and FCR under both TN and HS conditions with these effects being most pronounced over the latter stages of the growing period. Dietary POL reduced rectal temperature and respiration rate under HS and again these effects were more evident at the end of experiment [21,22]. The responses were also dose-dependent with the highest dose investigated being most efficacious. The positive effects of POL on regulating the expression of heat shock proteins [23] could help chickens to reduce their body temperature. Polyphenols are micronutrients with strong antioxidants properties and potential health benefits such as improving digestion issues and weight gain [24]. Furthermore, POL has protective effects that can be related to inhibition of glucose absorption [21], the ability to act as hormones and growth regulators [13] and they can act as an anti-inflammatory agents that prevent oxidative stress [10,12]. As a result, POL could reduce endogenous heat production thereby leading to lower rectal temperature and respiration rate. The positive effects of POL on lowering body temperature could allow chickens to utilise more ingested energy on growth performance rather than other metabolic pathways during HS.

Current genetically improved commercial broilers, such as the Ross-308, can gain weight faster than unimproved breeds, and in a shorter period of time, but this is not without consequences. These improved broiler chickens are more susceptible to HS because of higher endogenous heat production. In this regard, there is a negative relationship between susceptibility to HS and production traits in chickens [25,26]. Therefore, fast growing genotypes produce more heat, leading to higher mortality rate than slow-growing genotypes under thermal stress conditions [27,28]. Indeed, fast growing genotypes have a reduced ability to respond and adapt to environmental temperature changes such as HS.

Thyroid hormones play a vital role in growth, metabolic rate and oxygen consumption and any alteration in thyroid hormone secretion can cause metabolic disorders in various organs such as the kidney [29,30]. Furthermore, altered thyroid hormone concentrations can modify kidney function and reduce sodium, potassium and other electrolyte absorption [30]. The present results indicated an increase in plasma thyroid hormones in response to HS which is in contrast with our previously published results [17], but consistent with others studies [31,32]. However, the differences between the present results and other studies or our previous experiment could be related to differences in age, sex or breed of broiler chickens. This suggests that genetic selection for growth and age of chickens may affect how thyroid hormones respond to environmental temperature. The change of thyroid response in growth-selected birds could affect Ca^2+^ regulation in muscle and thus influence the post-mortem meat quality. Although there was no change in plasma Ca^2+^ concentrations during HS in the current experiment, the Ca content in skeletal muscle is regulated by thyroid hormones. Also, HS reduced plasma concentrations of potassium which participates in acid-base balance, maintenance of osmotic pressure, amino acid absorption and development of membrane potentials of cells [33]. Potassium does not function independently, and it is in relation with sodium concentrations that is necessary for optimal growth performance and amino acid metabolism [34]. Therefore, the reduced plasma concentrations of potassium, sodium and the osmolyte betaine during HS could impair growth performance and water content as indicated in the present results. In this regard, higher level of potassium during HS could increase thermotolerance in broiler chickens [35], leading to better performance. Interestingly, the level of potassium increased linearly with doses of POL under HS, suggesting that POL could help chickens to cope with HS.

Recently we characterised changes in distribution of the organic osmolyte betaine during heat stress in broilers. Supplementation with betaine resulted in increased distribution to skeletal muscle, reduced lipid peroxidation and improved meat tenderness [16,17]. The current study confirms the observation that HS reduces plasma and muscle betaine, likely because of increased betaine utilisation. Furthermore POL supplementation, at least at 2 and 4 mg/kg, appeared to improve muscle betaine concentrations, and this may have implications for improved meat quality.

The MFI is an important measure of ultrastructural changes that occur in myofibrils post-mortem and is positively correlated with meat tenderness [36,37]. In the present study there was decrease in MFI and an increase in shear force during HS which is consistent with the generation of excessive reactive oxygen species leading to the oxidation of myofibrillar proteins and a decrease in tenderness. Previously, we have shown that HS decreased MFI in chickens although in that study shear force was unaltered [17]. In contrast, others have found that HS increased shear force in chickens compared to their counterparts held under TN conditions [3]. Importantly, dietary POL reduced shear force and tended to increase MFI in a linear manner which is consistent with protection against the oxidative stress that occurs during HS.

Pale soft and exudative (PSE) meat may occur in chickens exposed to HS as a result of increased muscle glycolysis as demonstrated by the lower muscle pHu which may have led to paler meat with a lower water content than muscles from chickens reared under TN conditions [38,39]. Similarly, meat from chickens exposed to HS had lower pH, was paler, and had greater drip loss than muscles from chickens maintained under TN conditions [40]. Also, cyclic HS resulted in paler meat with a lower ultimate pH than that observed in chickens housed under TN conditions [41].

Heat stress increased oxidative damage to muscle tissues as determined by increased TBARS. Heat stress produces reactive oxygen species because of denaturation of proteins, resulting in more significant oxidative damages in tissues [42]. These results suggest that regardless of supplementation, HS is one of the major causes of oxidative stress in chickens and other farm animals muscle [43,44]. Increased lipid and protein oxidation can be determined by increased TBARS and microbiological growth, and colour deterioration for broiler chickens exposed to HS [45,46]. Importantly, higher doses of dietary POL were able to counteract the effects of HS on oxidation and even reduce oxidation under both temperature conditions.

Heat stress decreased breast muscle water content but had no main effect on drip or cooking loss. Previously, we found that HS decreased both water content and cooking loss with no effect on drip loss [17]. In another study, cooking loss was reduced after HS although the meat was paler with higher pHu [47] which is consistent with current observations. On the other hand, [17] found no effect of HS on pH or colour of breast muscle. As there is inconsistency between different studies regarding the effects of HS on the incidence of PSE meat in broiler chickens, other factors may have been involved. Importantly, dietary POL reversed many of the effects of HS on muscle colour, pH and water content.

## 5. Conclusions

The present data indicate that HS decreased growth performance and increased respiration rate and rectal temperature in broilers with resultant deleterious effects on meat quality. Supplemental POL was able to partially ameliorate the negative effects of HS on performance and meat quality as indicated by the reduction in muscle TBARS, MFI, shear force, colour and pH under both TN and HS conditions. The responses to POL were linear in nature with the greatest responses occurring at the highest dose investigated.

## Figures and Tables

**Table 1:** Polygain contents.

Radicals	ORAC (μmol TE/100 g) *
Peroxyl radicals	33,300
Hydroxyl radicals	162,400
Peroxynitrite	7300
Super oxide anion	45,100
Singlet oxygen	27,700
**Minerals**	**Per 100 g**
Sodium	50–200 mg
Potassium	2000–4000 mg
Selenium	<0.05 mg
Chromium	0.20–0.50 mg
Calcium	300–500 mg
Iron	10–15 mg
Magnesium	3000–5000 mg
Zinc	0.5–1.5 mg
**Nutritional Analysis**	**Per 100 g**
Energy	600 kJ
Protein	6.9 g
Total fat	<0.1 g
Carbohydrate	26.6 g

* Min 200,000 µmoles TE per 100 g (TE as Trolox equivalents) [15].

**Table 2:** Effect of dose (D) of dietary polygain on performance parameters when chickens exposed to thermoneutral (TN) and heat stress (HS) temperature (T) conditions.

		Dose of Polygain, g/kg						
		0	2	4	6	10	SED ^1^	Significance ^2,3^
**Daily body gain, g/d**								
0 to 21 days	TN	33.3	30.6	33.9	29.8	37.0	2.49	
	HS	31.8	33.8	31.4	31.4	32.8		
21 to 35 days	TN	84.0	88.4	88.8	94.0	96.4	5.04	T *, L **
	HS	80.1	82.8	86.3	89.0	87.6		
0 to 35 days	TN	53.9	53.7	55.9	55.5	60.4	2.46	T ^+^, L **
	HS	51.1	53.5	54.7	54.2	55.4		
**Daily feed intake, g/d**								
0 to 21 days	TN	48.7	52.6	52.1	52.2	48.0	5.2	
	HS	45.4	52.7	51.8	47.2	48.3		
21 to 35 days	TN	147	135	145	140	149	8.04	T *
	HS	128	147	135	128	136		
0 to 35 days	TN	87.9	85.5	89.3	87.5	88.4	5.05	T ^+^
	HS	78.5	90.2	85.0	79.7	83.3		
**Feed conversion ratio**								
0 to 21 days	TN	1.46	1.72	1.54	1.77	1.30	0.186	
	HS	1.45	1.56	1.68	1.51	1.50		
21 to 35 days	TN	1.76	1.53	1.64	1.50	1.55	0.098	L *
	HS	1.60	1.78	1.57	1.44	1.55		
0 to 35 days	TN	1.65	1.59	1.60	1.58	1.46	0.101	L *
	HS	1.54	1.69	1.61	1.46	1.53		
**Slaughter weight, g**	TN	1925	1917	1994	1978	2154	86.4	T ^+^, L **
	HS	1828	1912	1950	1936	1976		

^1^ Standard error of the difference for T × D. ^2^ Level of significance for main and interactive effects of T and linear (L) and quadratic (Q) effects of polygain. ^3^ + *p* < 0.10; * *p* < 0.05; ** *p* < 0.01; *** *p* < 0.001. Other main and interactive effects *p* > 0.10.

**Table 3:** Effect of dose (D) of dietary polygain on physiological responses and blood gas changes under thermoneutral (TN) and heat stress (HS) temperature (T) conditions.

		Dose of Polygain, g/kg						
		0	2	4	6	10	SED ^1^	Significance ^2,3^
Rectal temperature, °C	TN	41.6	41.3	41.3	41.3	41.4	0.13	T ***, L ***, Q *, T.L **
	HS	42.7	42.3	42.2	42.4	42.0		
Respiration rate, bpm	TN	66.0	64.0	62.0	63.0	64.0	8.20	T ***, L *, T.L *
	HS	177	163	160	154	148		
pH	TN	7.27	7.40	7.35	7.32	7.40	0.037	T ***, L ^+^
	HS	7.45	7.39	7.44	7.48	7.43		
pCO_2_, mm Hg	TN	55.6	41.5	54.6	55.4	50.7	4.73	T ***
	HS	40.1	45.3	42.5	35.0	42.4		
Total CO_2_, mM	TN	27.4	26.9	31.3	30.1	31.8	1.56	L ^+^, T.L *
	HS	29.8	29.0	29.8	26.9	29.5		
pO_2_, mm Hg	TN	30.6	45.6	36.7	42.0	29.3	4.11	Q ***
	HS	32.8	36.9	36.4	40.2	31.9		
O_2_ saturation,%	TN	48.6	81.2	65.6	70.7	54.1	7.59	Q ***
	HS	64.8	68.9	71.1	78.7	62.8		
HCO_3_, mM	TN	25.6	25.6	29.6	28.4	31.5	1.54	L *, T.L **
	HS	28.5	27.6	28.5	25.8	28.2		
Anion gap, mM	TN	20.8	19.0	16.0	17.7	14.3	0.92	T ***, L ***, T.L ***
	HS	15.0	15.3	14.7	16.7	16.0		
Base excess, mM	TN	-1.17	0.72	3.40	2.03	6.23	1.55	L **, T.L **
	HS	4.23	2.43	3.93	2.10	3.50		

^1^ Standard error of the difference for T × D. ^2^ Level of significance for main and interactive effects of T and linear (L) and quadratic (Q) effects of polygain. ^3^ + *p* < 0.10; * *p* < 0.05; ** *p* < 0.01; *** *p* < 0.001. Other main and interactive effects *p* > 0.10.

**Table 4:** Effect of dose (D) of dietary polygain on haematology, electrolytes, metabolites, plasma thyroid hormones and plasma and muscle betaine (*Pectoralis major*) during thermoneutral (TN) and heat stress (HS) temperature (T) conditions.

		Dose of Polygain, g/kg						
		0	2	4	6	10	SED ^1^	Significance ^2,3^
Haematocrit,%	TN	19.5	19.0	21.7	19.7	18.7	1.10	T ^+^, Q *
	HS	19.0	19.0	19.3	19.0	18.0		
Hgb ^4^, g/dL	TN	6.65	6.30	7.37	6.60	6.30	0.383	Q ^+^
	HS	6.40	6.37	6.60	6.47	6.10		
Potassium, mM	TN	6.78	6.49	6.43	6.33	5.97	0.46	T ***, T.L *
	HS	5.20	5.33	5.50	5.60	5.87		
Sodium, mM	TN	151	148	151	149	148	1.0	T ***
	HS	148	147	148	148	148		
Chloride, mM	TN	112	111	112	110	108	1.2	L *, T.L ^+^
	HS	110	109	110	111	109		
Calcium, mM	TN	1.52	1.40	1.50	1.52	1.46	0.043	Q ^+^, T.Q *
	HS	1.48	1.44	1.47	1.34	1.51		
Lactate, mM	TN	10.8	7.65	7.09	7.39	4.67	1.25	T ***, L **, T.L **
	HS	3.76	6.24	4.26	4.30	5.31		
Glucose, mM	TN	14.2	14.0	14.7	14.3	15.0	0.59	T ^+^
	HS	15.1	15.1	15.1	14.3	15.2		
T_3_^4^, pg/mL	TN	4.11	6.68	4.31	4.88	6.16	0.754	T.L ^+^
	HS	5.61	6.07	4.76	4.27	5.44		
T_4_^4^, pg/mL	TN	6.95	6.56	6.21	7.63	5.85	1.261	T ***
	HS	9.56	9.34	9.16	8.89	9.39		
Plasma betaine, µmol/L	TN	133	149	142	136	133	7.76	T ***, Q *
	HS	102	115	116	107	101		
Muscle betaine, µmol/g	TN	246	152	144	117	115	18.3	T **, L ***, Q **, T.L *** T.Q **
	HS	131	148	122	118	113		

^1^ Standard error of the difference for T × D. ^2^ Level of significance for main and interactive effects of T and linear (L) and quadratic (Q) effects of polygain. ^3^ + *p* < 0.10; * *p* < 0.05; ** *p* < 0.01; *** *p* < 0.001. Other main and interactive effects *p* > 0.10. ^4^ Haemoglobin (Hgb); triiodothyronine (T_3_); thyroxine (T_4_).

**Table 5:** Effect of dose (D) of dietary polygain on breast muscle quality (*Pectoralis major*) during thermoneutral (TN) and heat stress (HS) temperature (T) conditions.

		Dose of Polygain, g/kg						
		0	2	4	6	10	SED ^1^	Significance ^2,3^
Cooking loss,%	TN	22.8	21.6	23.0	20.9	22.4	1.28	
	HS	21.5	22.2	21.2	22.5	21.4		
Drip loss,%	TN	2.95	2.11	2.21	1.92	1.58	0.356	L **, T.L *
	HS	2.13	1.71	2.10	2.49	1.76		
Water content,%	TN	76.2	76.6	77.3	77.1	78.7	1.02	T *, L **
	HS	74.7	75.1	78.3	75.4	76.7		
Shear force, N	TN	24.1	20.1	20.9	19.9	19.4	1.61	T ***, L **
	HS	24.8	26.3	23.3	23.1	23.1		
MFI ^4^	TN	82.0	69.8	106	97.8	113	19.13	T **, L ^+^
	HS	64.9	60.0	86.0	69.1	68.7		
Carbonyl	TN	3.22	3.00	3.08	2.84	2.89	0.403	Q ^+^
nmol/mg protein	HS	3.77	3.45	2.94	2.90	3.35		

^1^ Standard error of the difference for T × D. ^2^ Level of significance for main and interactive effects of T and linear (L) and quadratic (Q) effects of polygain. ^3^ + *p* < 0.10; * *p* < 0.05; ** *p* < 0.01; *** *p* < 0.001. Other main and interactive effects *p* > 0.10. ^4^ Myofibrillar fragmentation index.

**Table 6:** Effect of dose (D) of dietary polygain and time post-slaughter (TM) on chicken breast (*Pectoralis major*) colour and pH after being housed thermoneutral (TN) and heat stress (HS) temperature (T) conditions.

			Dose of Polygain, g/kg						
	Time (TM)		0	2	4	6	10	SED ^1^	Significance ^2,3^
L*	24 h	TN	55.5	53.0	56.3	56.3	55.2	1.95	TM ***, T *
		HS	55.7	55.3	60.3	56.2	56.3		
	72 h	TN	50.1	47.5	49.8	51.4	51.8		
		HS	50.6	50.9	53.1	50.8	51.7		
a*	24 h	TN	1.99	1.99	1.82	2.06	2.38	0.420	TM **, T ***
		HS	1.54	1.26	1.42	1.19	1.29		
	72 h	TN	1.55	2.21	1.49	1.26	1.79		
		HS	0.69	1.29	1.29	1.21	0.62		
b*	24 h	TN	6.07	5.60	6.25	6.79	6.26	0.606	TM ***, L *, Q *, T.L *
		HS	5.11	6.01	6.50	6.44	6.00		
	72 h	TN	4.46	3.97	4.43	5.21	5.53		
		HS	4.50	4.28	6.09	4.74	4.31		
pH	10 min	TN	6.77	6.88	6.88	6.90	6.90	0.112	T ***, TM ***, L ***, T.TM ***
		HS	6.83	6.78	6.86	6.84	6.99		TM.L *, T.TM.L *
	1 h	TN	6.44	6.72	6.64	6.75	6.60		
		HS	6.60	6.49	6.64	6.58	6.67		
	24 h	TN	5.72	5.91	5.98	6.15	6.33		
		HS	5.61	5.60	5.86	5.81	5.73		

^1^ Standard error of the difference for T × D. ^2^ Level of significance for main and interactive effects of T and linear (L) and quadratic (Q) effects of polygain. ^3^ + *p* < 0.10; * *p* < 0.05; ** *p* < 0.01; *** *p* < 0.001. Other main and interactive effects *p* > 0.10.
